# Acute gallbladder torsion: an unexpected intraoperative finding

**DOI:** 10.1186/1749-7922-3-9

**Published:** 2008-02-22

**Authors:** Gnananandan Janakan, Abraham A Ayantunde, Happy Hoque

**Affiliations:** 1 Department of Surgery, Queen Mary's Hospital, Sidcup, Kent, DA14 6LT, UK

## Abstract

Gallbladder torsion is an uncommon clinical entity and a difficult condition to diagnose preoperatively. Since its first description in 1898 by Wendel there have been over 500 documented cases in the literature. It is known to occur when there is rotation of the gallbladder along the axis of the cystic duct and vascular pedicle. Except for isolated cases reported in childhood, this disease is more frequently encountered in the elderly with 85% of the cases reported between the ages of 60 and 80 years. There is a female preponderance with a female to male ratio of 3:1. Gallbladder torsion typically presents as an acute abdomen requiring emergency surgery but preoperative diagnosis of gallbladder torsion is difficult and most cases are found as a surprise at surgery.

We report a case of acute gallbladder torsion in an elderly lady and review the clinical aspect of the disease.

## Background

Gallbladder torsion is an uncommon clinical entity and a difficult condition to diagnose preoperatively. It is known to occur when there is rotation of the gallbladder along the axis of the cystic duct and vascular pedicle. Since its first description in 1898 by Wendel, there have been over 500 documented cases in the literature [1,2]. Except for isolated cases reported in childhood, this disease is more frequently encountered in the elderly with 85% of the cases reported between the ages of 60 and 80 years [2-5]. There is a female preponderance with a female to male ratio of 3:1 [2-5].

The common causative factor is an abnormality in the anatomy of the gallbladder and its associated vascular pedicle. In general, this is mainly due to the two mesenteric anatomical variations including either a wide or a long mesentery with cystic artery and duct. This is referred to as a 'floating' or 'pedunculated' gallbladder [2,3,5-7]. In the elderly, loss of viscera fat with liver atrophy can result in acquired long mesentery [7]. Gallbladder torsion typically presents as acute abdomen requiring emergency surgery but preoperative diagnosis of gallbladder torsion is difficult and most cases are found as a surprise at surgery.

We report a case of acute gallbladder torsion in an elderly lady and review the clinical aspects of the disease.

## Case presentation

A 79 year old lady presented to Accident & Emergency department with a two day history of sudden onset colicky abdominal pain associated with vomiting and progressive abdominal distension. She had failed to pass any faeces or flatus 24 hours prior to presentation. She denied any history of recent change in bowel habit or weight loss. There was no significant relevant past medical history.

She was dehydrated at presentation with the following vital signs: HR-107 b/min, BP-119/50 mmHg, T-36.5°C, oxygen saturation-97% on air. Abdomen was distended and resonant to percussion with a palpable tender central abdominal mass around the umbilicus measuring 5 cm by 6 cm. Bowel sounds and digital rectal examinations were normal. Erect chest X-ray was unremarkable, whilst abdominal X-ray showed normal small bowel gas pattern and right sided colonic faecal loading. Arterial blood gas on air showed pH 7.49, HCO_3 _25.1 & BE +2.3. Laboratory blood tests revealed mildly deranged liver function tests (LFT) [Bilirubin 25 μmol/L, ALP 276 U/L, ALT 277 U/L & GGT 301 U/L], C-reactive protein (CRP) 152 mg/L and white blood cell (WBC) count 20 × 10^9 ^(neutrophils 17.8 × 10^9^). She was admitted with the provisional diagnosis of acute large bowel obstruction secondary to a possible transverse colon tumour. Differential diagnoses of acute cholecystitis and mesenteric cyst torsion was entertained.

**Figure 1:**
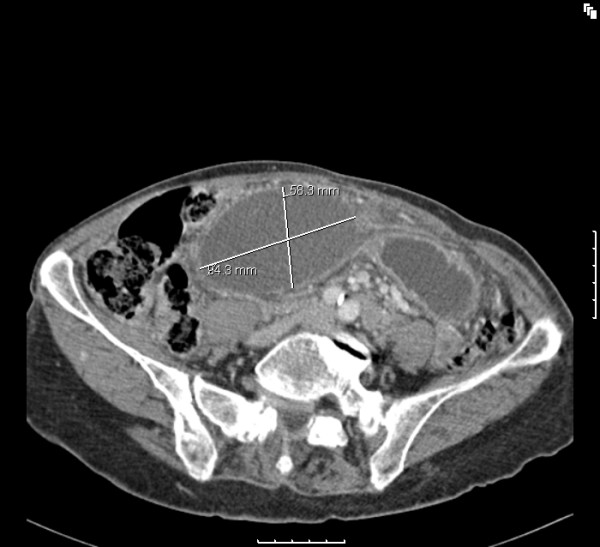
CT scan of the abdomen in horizontal section showing a cystic mass.

**Figure 2:**
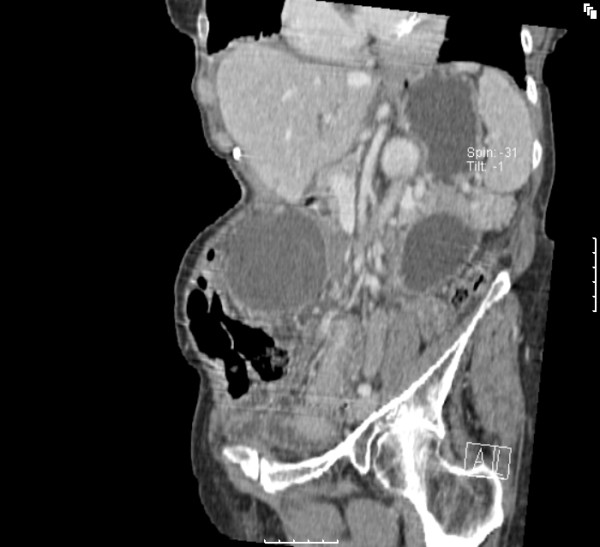
CT scan of the abdomen and pelvis in sagittal section.

She was resuscitated with IV fluids and an emergency computerized tomography (CT) scan of the abdomen and pelvis was performed. This was reported as showing an oval cystic lesion in the central abdomen, with an AP diameter of 6 cm and a transverse diameter of 9 cm. This structure was unilocular with no connection to the bowel or mesentery but compressing the transverse colon (Figures 1 &2). There were dilated small bowel loops in the pelvis. The liver, spleen, pancreas and both kidneys were normal. The decision to continue resuscitation and to further evaluate the patients for the nature of this mass was taken. However, by the next day, her pain had increased in severity and the central abdominal mass was more tender. The decision was then taken to proceed to an emergency laparotomy.

**Figure 3:**
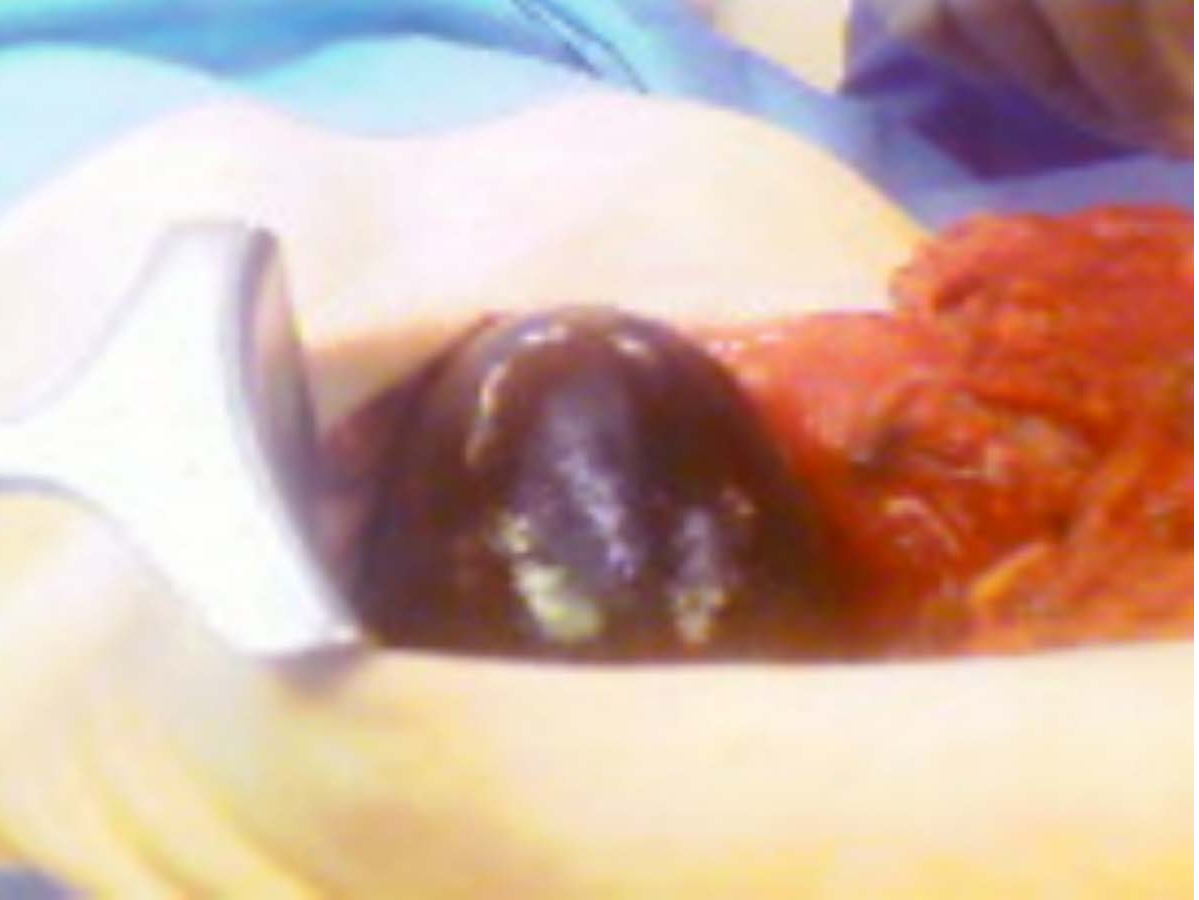
Intraoperative finding of gangrenous gallbladder.

**Figure 4:**
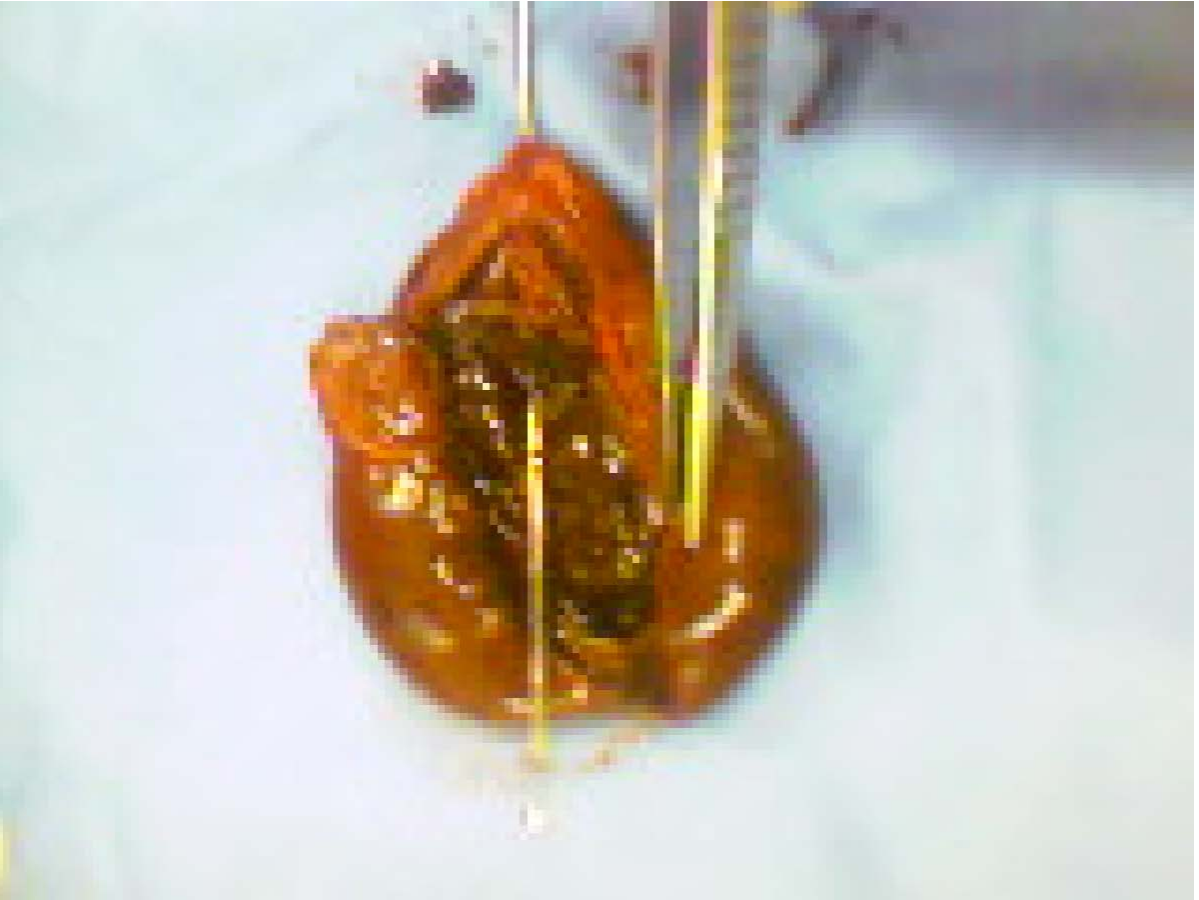
Opened gangrenous gallbladder.

Emergency laparotomy through a midline incision was performed and findings at operation included a large, gangrenous gallbladder with a 360 degree clockwise torsion on its long pedicle. The gallbladder was distinctly suspended free from the liver edge and lying over the greater omentum and transverse colon with some haemorrhagic peritoneal fluid reaction. The torted pedicle was undone and cholecystectomy was performed. The opened gallbladder had complete hemorrhagic infarct with wall thickness of about 1 cm across (Figures 3 &4). The pathological examination of the specimen revealed an 8 cm enlarged gallbladder, 4.5 cm in diameter, with a 1 cm thick oedematous wall with mucosal congestion and the histological findings were consistent with acute gangrenous cholecystitis with extensive necrosis of the wall. Post-operative recovery was uneventful and she was discharged home 8^th ^day post surgery. The delay in discharge was due to social reason.

## Discussion

Torsion of the gallbladder is a rare clinical condition of the hepatobiliary system, with a reported clinical incidence of 1 in 365,520 hospital admissions [6,8,9]. In fact, less than 50 cases have been reported in the literature within the last 30 years [10]. Although it mainly affects the elderly especially women, it has been reported in all age groups [2-4,8,9]. Kitagawa et al [11] in 1997 reported two cases of acute gallbladder torsion in boys aged 4 and 5 years who presented with acute abdominal pain.

The aetiology of gallbladder torsion is unknown; however, there are several factors that have been postulated as playing significant causative roles. There are two requirements for developing gallbladder torsion, the presence of a long mesentery or a very short/absent mesentery allowing mobility of the gallbladder along its vertical axis and the other factor is generalized loss of elastic tissue and visceroptosis in the elderly. These factors contribute to non-fixation of the gallbladder to the inferior margin of the liver causing the gallbladder to hang freely in the peritoneal cavity [2,3,5-8,10]. Other precipitating factors include increased or violent perilstasis of the surrounding stomach, duodenum and colon, presence of scoliokyphosis, arterosclerosis of the cystic artery [4,7,8]. The presence of gallstone does not seem to be a strong risk factor as it is only reported in 25–50% of the cases [5,8]. The gallbladder torsion may be complete through a 360 degree resulting in strangulation of the vascular supply and gangrenous cholecystitis or incomplete through about 180 degree and this causes intermittent symptoms of biliary colic [3,5,12]. The direction of torsion may be clockwise or anticlockwise and both directions are reported with equal incidence [4,12].

The symptoms of gallbladder torsion are largely non-specific and this makes preoperative diagnosis difficult on the basis of history and physical examinations alone [8,9]. Patient typically presents with acute onset abdominal pain with or without vomiting. There may be the presence of a tender mobile mass indicating a 'floating gallbladder'. Our patient presented atypically with features of bowel obstruction and a tender mass around the umbilicus. Lau et al [13] proposed triple triads for a clinical diagnosis of gallbladder torsion, which include specific symptoms (short history, abdominal pain and early vomiting), physical signs (abdominal mass, absence of toxaemia and pulse rate-temperature discrepancy) and patient's physical characteristics (thin, elderly and deformed spine). The clinical features may mimic that of acute cholecystitis but low frequency of fever and jaundice, absence of toxaemia and poor response to antibiotic therapy can help differentiate it from cholecystitis [14].

Laboratory investigations are largely unhelpful. Raised white blood cell count and C-reactive protein are frequent findings especially with the onset of gangrenous cholecystitis. Liver function tests are usually normal in patients with gallbladder torsion [12]. Imaging investigations like ultrasound and Computerised tomography (CT) may complement the diagnosis of gallbladder torsion but are sometime non-specific [3,14]. Ultrasound scan will normally identify the enlarged gallbladder inferior to its normal anatomical position with a thickened wall and surrounded by free fluid [9].

Kitagawa H et al [11] provided CT criteria for identifying gallbladder torsion and this include fluid collection between gallbladder and gallbladder fossa of the liver, the presence of horizontal rather than vertical axis of the gallbladder, the presence of a well-enhanced cystic duct located on the right side of the gallbladder and signs of inflammation such as oedema and thickening of the wall. Magnetic resonance (MR) imaging findings include a high signal intensity within the gallbladder wall on T1-weighted images suggesting haemorrhagic infarct and necrosis [9]. The presence of a large anteriorly floating gallbladder away from normal anatomical location with gross wall thickening and without gallstones in an elderly woman with kyphosis can be suggestive of gallbladder torsion.

Early diagnosis of gallbladder torsion is important in order to avoid the complication of perforation and the attendant bilious peritonitis. Early diagnosis and prompt surgical interventions have been shown to reduce the mortality to less than 5% [5,8]. Emergency cholecystectomy is required for gallbladder torsion. Previously, this condition was largely treated with open surgery but with increasing experience in laparoscopic cholecystectomy, laparoscopic approach is now recommended as the first choice. Laparoscopic approach has the benefit of confirming the diagnosis and early postoperative recovery. The principles of this approach include decompression, derotation, cholecystectomy with or without intraoperative cholangiogram [2,14,15]. Our patient had an open surgery because of the preoperative misdiagnosis of a transverse colonic mass causing bowel obstruction. In retrospective, a laparoscopic approach would have been a better option in treating this patient. The presence of a long mesentery and the separation of the gallbladder from its liver bed usually makes laparoscopic approach much easier than is the case with the ordinary cholecystectomy.

## Conclusion

Gallbladder torsion is rare and therefore requires a high index of suspicion for early preoperative diagnosis and prompt surgical intervention. This diagnosis should be considered in the setting of an elderly woman with atypical or non-resolving symptoms and signs of acute cholecystitis in spite of the use of adequate antibiotic therapy. Increasing incidence of gallbladder torsion is being encountered today and this is probably due to unreserved use of imaging investigations and laparoscopy. Early diagnostic imaging investigations and prompt cholecystectomy is the aim in order to achieve best patient outcome.

## Competing interests

The author(s) declare that they have no competing interests.

## Authors' contributions

GJ participated in the admission and the care of this patient, the conception, design, data collection and interpretation, manuscript preparation and literature search. AAA participated in the admission and the care of this patient, the conception, design, data collection and interpretation, manuscript preparation and literature search. HH participated in the admission and the care of this patient, the conception, design, data collection and interpretation, manuscript preparation and literature search. All authors read and approved the final manuscript.
